# Resveratrol ameliorates oxidative stress, inflammatory response and lipid metabolism in common carp (*Cyprinus carpio*) fed with high-fat diet

**DOI:** 10.3389/fimmu.2022.965954

**Published:** 2022-11-03

**Authors:** Di Wu, Jinnan Li, Ze Fan, Liansheng Wang, Xianhu Zheng

**Affiliations:** Key Laboratory of Aquatic Animal Diseases and Immune Technology of Heilongjiang Province, Heilongjiang River Fisheries Research Institute, Chinese Academy of Fishery Sciences, Harbin, China

**Keywords:** resveratrol, high-fat diet, oxidative stress, inflammatory response, lipid metabolism

## Abstract

High-fat diet is regarded as crucial inducers of oxidative stress, inflammation, and metabolic imbalance. In order to investigate the ameliorative potential of resveratrol against the progression of liver injury towards steatohepatitis, common carp (*Cyprinus carpio*) were distributed into six experimental groups and were fed with a normal-fat diet, a high-fat diet, and supplemented with resveratrol (0.8, 1.6, 2.4, and 3.2 g/kg diet) for 8 weeks. The high-fat diet decreased the antioxidant capacities, as well as causing the inflammatory response and lipid deposition of common carp. Resveratrol induced a marked elevation in the final body weight, weight gain rate, condition factor and significant decrease in the feed conversion ratio. Moreover, dietary resveratrol showed a significant decrease in the alanine aminotransferase, aspartate aminotransferase, triglyceride and low-density lipoprotein levels, which was accompanied by an increase in high-density lipoprotein concentration in serum. A significant elevation in total superoxide dismutase, catalase, glutathione peroxidase and a decreased malondialdehyde content were observed, along with a substantial elevation in antioxidant activities were found. Additionally, fish fed with resveratrol had an up-regulation of hepatic catalase, copper, zinc superoxide dismutase, glutathione peroxidase 1a, and glutathione peroxidase 1b gene expression *via* Nrf2 signaling pathway. Expectedly, our results also demonstrated that resveratrol regulates hepatic lipid metabolism in fish by inhibiting the expression of hepatic lipogenesis genes (acetyl-CoA carboxylase 1, fatty acid synthase, and sterol regulatory element binding protein 1), fatty acid uptake-related genes of lipoprotein lipase, and β-oxidation-related genes *via* PPAR-γ signaling pathway. Furthermore, dietary resveratrol reduced inflammation, as evident by down-regulating the interleukin-1β, interleukin-6, interleukin-8, and tumor necrosis factor-α expression levels and upregulating the interleukin-10 and transforming growth factor-β2 expression levels *via* NF-κB signaling pathway. As a whole, our results demonstrated that resveratrol defensed the impacts against high-fat diet on the serum biochemical, hepatic antioxidants, inflammation, and lipid metabolism.

## Introduction

Lipids are considered essential nutrients for aquatic animals, providing fish with the energy needed for survival. In global aquaculture, high-fat (HF) diets are widely used as an alternative energy source to protein (1). However, excessive lipids in the diet cause negative effects on slow growth, low immunity, oxidative stress and hepatic steatosis in cultured fish, which affect their health. Previous studies have confirmed that dietary HF diet reduces the growth performance, feed intake and feed efficiency in grass carp (*Ctenopharyngodon idella*) (2). In addition, excess lipids can also cause oxidative stress in organisms, that is, an imbalance between the production of reactive oxygen species (ROS) and the antioxidant defenses of organisms. Although antioxidants and enzymes with antioxidant activities such as superoxide dismutase (SOD), catalase (CAT), glutathione peroxidase (GSH-Px) and glutathione (GSH) constitute the defense system against ROS, the overloaded ROS will weaken the defense system and lead to oxidative stress (3). A study has shown that dietary HF diet to turbot (*Scophthalmus maximus*) remarkably decreased the catalase (CAT) and superoxide dismutase (SOD) activities as well as increasing the malondialdehyde (MDA) content in liver (4). Moreover, oxidative stress induces the cell damage and deregulated production of adipocytokines that contribute to dyslipidemia and liver steatosis. Growing evidence suggests that increased oxidative stress is involved in the pathogenesis of metabolic disease (5). Studies also confirmed that HF diet induced the metabolic inflammation and oxidative stress on blunt snout bream (*Megalobrama amblycephala*) (6) and yellow catfish (*Pelteobagrus fulvidraco*), resulting in metabolic dysbiosis (7). So far, preventing and treating oxidative stress, inflammation and metabolic diseases caused by an HF diet remains a major challenge for the global fish production.

Resveratrol (RSV) is a polyphenol found in many plants such as grapes and berries (8). The excellent antioxidant and anti-inflammatory activities of RSV have been demonstrated in multiple species. In human, RSV exhibits a wide spectrum of potential therapeutic activities against obesity, inflammation and oxidative stress (9, 10). Early studies (*in vitro* and *in vivo*) also proved that RSV might have regulatory effects on lipid metabolism in mice and rats (11, 12). In addition, accumulating evidence has demonstrated that RSV has beneficial effects in fish: for instance, RSV decreased the production of oxygen free radicals and lipid peroxides, protected the intact antioxidant capacity of cell membrane structure and function, and improved the antioxidant function of Nile tilapia (*Oreochromis niloticus*) (13). RSV has also been suggested to effectively reduce the increase of body fat and intraperitoneal fat in blunt snout bream, and slow down the occurrence of hepatic steatosis (14). As for the antioxidant mechanism of RSV, studies have shown that RSV is an activator of Nrf2, which can regulate the transcription of downstream related antioxidant genes through Nrf2 signaling pathway and inhibit the stress oxidation of organisms (15). In addition, it showed that RSV promoted the fatty acid synthesis in liver in the absence of fish oil in rainbow trout (*Oncorhynchus mykiss*) (16) and Pacific salmon (*Atlantic salmon*) (17). However, limited information about the protective effect and lipid metabolism mechanism of RSV on liver injury in common carp (*Cyprinus carpio*) induced by an HF diet is available.

Considering the critical role of the excellent antioxidant and anti-inflammatory activities of RSV, therefore, we hypothesized that RSV supplementation might protect the hepatic functions against the HF diet on oxidative stress, inflammation, and lipid metabolism. The main goal of this study was to identify the potential protective effects and mechanism of RSV against liver damage caused by an HF diet. Our data revealed that dietary RSV supplementation ameliorated the growth performance, hepatic oxidative stress, inflammatory response, as well as lipid metabolism in common carp fed with an HF diet.

## Materials and methods

### Experimental ingredients and diets

RSV (≥ 98% purity) was obtained from Jingzhu Biotechnology Co., Ltd. (Nanjing, China). Six experimental diets were prepared, including a normal fat diet (5.0% lipid, NF), an HF diet (15.0% lipid, HF), and the HF diet supplemented with 0.8 g/kg RSV (HFR8), 1.6 g/kg RSV (HFR16), 2.4 g/kg RSV (HFR24), and 3.2 g/kg RSV (HFR32). The basal diet formulations and analyzed proximate compositions of NF and HF diets are shown in **Table 1**. Glucose was used to balance the addition of RSV. The feed ingredients were ground into fine powder through an 80 mesh filter. All ingredients were thoroughly mixed with fish oil, soybean oil, and soybean lecithin. The mixture was mixed with water and then made into 2 mm diameter pellets. Subsequently, the pellets were dried in a warm air cabinet and then stored at -20°C until feeding.

**Table 1:** Formulation and proximate composition of experimental diets (% dry matter).

Ingredients (%)	NF	HF	Proximate compositions	NF	HF
Fish meal^a^	10.00	10.00	Crude protein^d^ (%)	31.80	31.70
Soybean meal^a^	50.00	50.00	Crude lipid^d^ (%)	4.96	14.85
Wheat middling^a^	20.00	20.00	Crude ash^d^ (%)	7.18	6.70
Fish oil	1.50	1.50			
Soybean oil	2.00	11.50			
Soybean lecithin	1.00	1.00			
Vitamine premix^b^	0.30	0.30			
Mineral premix^c^	0.20	0.20			
Choline chloride	0.50	0.50			
Ca(H_2_PO_4_)_2_	2.00	2.00			
Cellulose	9.90	0.40			
Carboxymethylcellulose sodium	1.00	1.00			
Lysine	0.20	0.20			
Methionine	0.40	0.40			
Glucose	1.00	1.00			
Total	100.00	100.00			

^a^Fish meal, obtained from Hefeng feedstuffs Co., Ltd, crude protein 600.00 g/kg, crude lipid 48.50 g/kg. Soybean meal, obtained from Hefeng feedstuffs Co., Ltd, crude protein 440.00 g/kg, crude lipid 15.00 g/kg. Wheat middling, obtained from Jinlongyu Grain, Oil and Food Co. Ltd, crude protein 130.00 g/kg, crude lipid 12.00 g/kg.

^b^The vitamin premix provided the following per kg of the diet: VA 8,000 IU, VC 500 mg, VD_3_ 3,000 IU, VE 60 mg, VK_3_ 5 mg, VB_2_ 30 mg, VB_6_ 15 mg, VB_12_ 0.5 mg, choline chloride 5,000 mg, nicotinic acid 175 mg, D-biotin 2.5 mg, inositol 1,000 mg, folic acid 5 mg, pantothenic acid 50 mg.

^c^The mineral premix provided the following per kg of the diet: Zn 25 mg, Cu 3 mg, Fe 25 mg, Mn 15 mg, I 0.6 mg, Co 0.1 mg, Se 0.4 mg.

^d^Crude protein, crude lipid, and crude ash were measurement value.

### Animals and growth trial

Common carp were obtained from a commercial aquafarm (Chengdu, China). Prior to the formal experiment, all carp were acclimated to experiment conditions and fed the control experimental diet (NF diet) for 2 weeks. After a 24 h fasting period, fish of similar sizes (1.18 ± 0.03 g) were randomly distributed into 18 tanks (0.7 m × 0.7 m × 0.8 m) with three replicates per treatment and 30 carp per tank. Each tank was provided with a continuous flow of water and continuous aeration through air stones to maintain dissolved oxygen at near saturation. Each diet was randomly assigned to triplicate tanks. Fish were fed three times per day (08:00 am, 13:00 pm and 17:00 pm) at approximately 5% of fish weight for 8 weeks. During the experiment, the fish were kept in natural light and dark cycle. Water temperature was maintained at 24 ± 1.0°C, pH at 7.6 ± 0.4, and dissolved oxygen > 6.0 mg/L. One third water in the aquarium was changed every three days, and fish feeding and mortality were recorded daily.

### Sample collection

Fish were fasted for 24 h before sampling, and all fish in each tank were counted and weighed. Subsequently, 6 fish in each tank (a total of 18 fish per group) were randomly selected and anesthetized with 100 mg/L tricaine methane sulfonate (MS-222, Sigma, St. Louis, MO). Blood samples were obtained from the caudal vein of 6 fish from each tank with 1 ml syringes and allowed to clot at 4°C for 6 h, closely followed by centrifuging at 3500 g for 10 min. The serum was collected and then stored at -80°C to analyze. After sacrifice, the viscera (including liver, gallbladder and intestine) were quickly removed. Viscera and liver separately from 6 fish in each tank were weighed for subsequent biometric parameter calculation. After this, the livers of 3 fish were snap-frozen in liquid nitrogen then stored at -80°C for quantitative real-time PCR, and the livers of another 3 fish were collected and then stored at -20°C for subsequent antioxidant and enzyme activity analysis.

### Biochemical analysis

For hematological functions parameters, alanine aminotransferase (ALT), aspartate aminotransferase (AST), total triglycerides (TG), total cholesterol (TC), high-density lipoprotein (HDL), and low-density lipoprotein (LDL) were determined by an automatic biochemical analyzer (Olympus AU400, Olympus Optical Co., Ltd., Japan) using the corresponding commercial kits purchased from 3V Bioengineering Co., Ltd., Weifang, China.

For digestive enzyme assay, lipase (LPS), amylase (AMS), and trypsin (Try) activities were analyzed using commercial assay kits (Nanjing Jiancheng Bioengineering Institute, Nanjing, China) according to the manufacturer’s instructions.

For antioxidant-related parameters assay, liver homogenates were prepared according to the kit instructions. Total superoxide dismutase (T-SOD), catalase (CAT), glutathione (GSH), glutathione peroxidase (GSH-Px), as well as malondialdehyde (MDA) were determined using the commercial kits (Nanjing Jiancheng Bioengineering Institute) according to the manufacturer’s instructions.

### Quantitative real-time PCR analysis

The total RNA from liver was extracted using an RNAiso Plus kit (Takara, Dalian, China). After determining the concentration and purity of the total RNA, cDNA was synthetized using a PrimeScriptTM RT reagent Kit (Takara, Dalian, China). Conclusively, quantitative real-time PCR was performed using an ABI 7500 real-time PCR System (ABI, Applied Biosystems, USA) with SYBR Green (Takara, Dalian, China), which was described previously (18). All specific primers for qPCR are shown in **Table 2**. After normalization against the reference gene β-actin, the relative levels of mRNA expressions were calculated according to the 2^-△△Ct^ method (19).

**Table 2:** Primer sequences for quantitative real-time PCR.

Genes	Forward (5’–3’)	Reverse (5’–3’)	Accession number
CAT^a^	CTGGAAGTGGAATCCGTTTG	CGACCTCAGCGAAATAGTTG	GQ376154.1
CuZnSOD^b^	TGGCGAAGAAGGCTGTTTGT	TTCACTGGAGACCCGTCACT	LN596307.1
GPx1a^c^	GTGACGACTCTGTGTCCTTG	AACCTTCTGCTGTATCTCTTGA	GQ376155.1
GPx1b^d^	TATGTCCGTCCTGGCAATGG	ATCGCTCGGGAATGGAAGTT	JF411606.1
Nrf2^e^	TTCCCGCTGGTTTACCTTAC	CGTTTCTTCTGCTTGTCTTT	JAEOAB010000029
ACC1^f^	GTCACTGGCGTATGAGGATATT	TGTGTAGAAGTTGCTGTTGACCA	LN590687.1
FAS^g^	GACAGGCCGCTATTGCTATT	TGCCGTAAGCTGAGGAAATC	GQ466045.1
SREBP1^h^	CGTCTGCTTCACTTCACTACTC	GGACCAGTCTTCATCCACAAA	LN590913.1
PPAR-γ^i^	CTTCGTGAACCTGGACTTG	ATCTGACCGTAGGAGATGAG	FJ849064.1
RXR-α^j^	CCGCAACGAGAACGAGGTG	AGGGCATCGGGACATTGGT	LN590693.1
LPL^k^	GCAGATGCCCAAAGCACTCTTTC	GTTCTTGCGGCAGCTGAGACA	KM213240.1
IL-1β^m^	AACTTCACACTTGAGGAT	GACAGAACAATAACAACAAC	KC008576
IL-6^n^	GACCAGCAGGTACGTCTCAACAC	TCCTTCATACGCCGTCATGTTCAC	LN590906.1
IL-8°	AAACTGAGAGTCGACGCATTG	TTTTCAATGACCTTCTTAACCCAG	EU011243.1
TNF-α^p^	AAGTCTCAGAACAATCAGGAA	TGCCTTGGAAGTGACATT	AJ311800
IL-10^q^	GCCAGCATAAAGAACTCG	CCAAATACTGCTCGATGT	JX524550.1
TGF-β2^r^	GGGACATCATCGCCATCT	TGACATTCTCGGCAGGGT	JAEOAB010000023.1
NF-κB^s^	TATTCAGTGCGTGAAGAAG	TATTAAAGGGGTTGTTCTGT	LN590678.1
MyD88^t^	AAGAGGATGGTGGTAGTCA	GAGTGCGAACTTGGTCTG	LN590716.1
β-actin	GATCGGCAATGAGCGTTTCC	ACGGTGTTGGCATACAGGTC	M24113.1

^a^CAT, catalase;

^b^CuZnSOD, copper, zinc superoxide dismutase;

^c^GPx1a, glutathione peroxidase 1a;

^d^GPx1b, glutathione peroxidase 1b;

^e^Nrf2, nuclear factor erythroid 2-related factor 2;

^f^ACC1, acetyl-CoA carboxylase 1;

^g^FAS, fatty acid synthase;

^h^SREBP1, sterol regulatory element binding protein 1;

^i^PPAR-γ, peroxisome proliferator activated receptor γ;

^j^RXR-α, retinoid X receptor;

^k^LPL, lipoprotein lipase;

^m^IL-1β, interleukin-1β;

^n^IL-6, interleukin-6;

^o^IL-8, interleukin-8;

^p^TNF-α, tumor necrosis factor-α;

^q^IL-10, interleukin-10;

^r^TGF-β2, transforming growth factor β2;

^s^NF-κB, nuclear factor κB;

^t^MyD88, myeloid differentiation factor 88.

### Statistical analysis

Data from each fish in the same replicate were averaged and used for subsequent analyses. Students’ *t*-test was used to compare the data between the NF and HF groups. One-way analysis of variance (ANOVA) followed by Tukey’s multiple-range tests was used for analyzing the data among different RSV levels in the HF diet. The linear or quadratic effect of RSV were assayed by orthogonal polynomial contrasts using the SPSS 22.0 software. All data are presented as the means ± standard error of the mean (SEM). In all statistical tests used, *P* < 0.05 was considered significantly different. Data visualization was analysed with the GraphPad Prim 9.0 (GraphPad Inc., La Jolla, CA, USA).

## Results

### Growth performance

The growth performance of common carp fed with RSV supplementation are presented in **Table 3**. The final body weight (FBW) and weight gain rate (WGR) in fish fed with HF treatments were remarkably lower than those in the NF group (*P* < 0.01 or *P* < 0.001), whereas an opposite result was noted in hepatosomatic index (HSI) (*P* < 0.001). The FBW, WGR, and condition factor (CF) increased linearly (*P* < 0.05 or *P* < 0.01) in response to increasing RSV levels. Notably, the feed conversion ratio (FCR) decreased linearly (*P* < 0.05) as RSV levels increased in the diet. However, no significant difference of the HSI was appeared among all the RSV treatments (*P* > 0.05).

**Table 3:** Effects of resveratrol supplementation on the growth performance in common carp (*Cyprinus carpio*)^a^.

	NF	HF	HFR8	HFR16	HFR24	HFR32	Polynomial contrasts^b^	
							Linear	Quadratic
FBW^c^ (g)	10.40 ± 0.06	9.56 ± 0.02^b**^	9.94 ± 0.08^ab^	10.23 ± 0.36^ab^	10.10 ± 0.07^ab^	10.41 ± 0.20^a^	0.006	0.915
WGR^d^ (%)	777.67 ± 7.23	711.00 ± 15.11^b***^	764.33 ± 36.12^ab^	740.67 ± 32.81^ab^	750.33 ± 18.77^ab^	781.67 ± 29.56^a^	0.030	0.852
FCR^e^	1.35 ± 0.06	1.45 ± 0.04^a^	1.34 ± 0.06^b^	1.39 ± 0.04^ab^	1.37 ± 0.03^ab^	1.33 ± 0.06^b^	0.034	0.518
HSI^f^ (%)	8.30 ± 0.82	9.56 ± 0.83^***^	9.63 ± 1.12	8.13 ± 1.73	9.54 ± 2.10	9.63 ± 1.61	0.077	0.803
CF^g^ (g/cm^3^)	1.44 ± 0.15	1.45 ± 0.12^ab^	1.43 ± 0.14^b^	1.45 ± 0.18^ab^	1.51 ± 0.19^a^	1.49 ± 0.10^ab^	0.042	0.522

^a^Values expressed as mean ± SEM, n = 3. “^*^” represented statistical difference between the NF group and the HF group using student’s t-test (^**^P < 0.01 or ^***^P < 0.001). “^ab^” represented statistical difference among different RSV levels in the HF diet. Values in the same row with different superscripts mean significant difference (P < 0.05)

^b^P-values indicateed a linear and quadratic to dietary RSV levels using orthogonal polynomial analysis.

^c^FBW, final body weight.

^d^WGR, weight gain rate = (final body weight - initial body weight)/initial body weight × 100%.

^e^FCR, feed conversion ratio = wet weight gain (g)/dry feed intake (g).

^f^HSI, hepatosomatic index = final liver weight (g)/final body weight (g) × 100%.

^g^CF, condition factor = final body weight(g)/final body length^3^ (cm) × 100%.

### Hematological functions parameters

**Figure 1:**
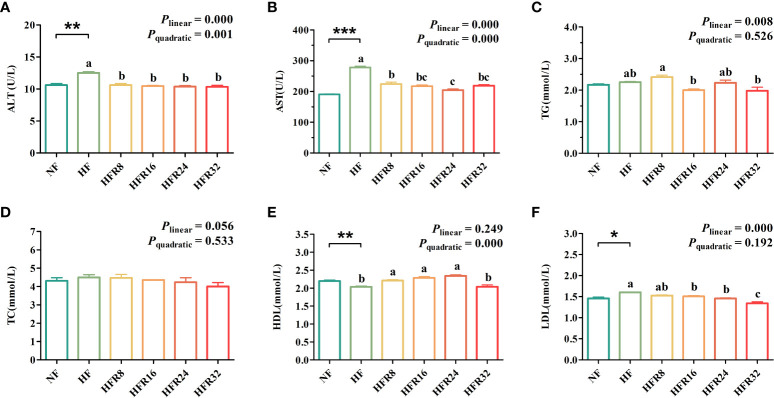
Effects of resveratrol supplementation on the hematological functions parameters in common carp *(Cyprinus carpio*). **(A)** alanine aminotransferase (ALT) activity; **(B)** aspartate aminotransferase (AST) activity; **(C)** triglyceride (TG) concentration; **(D)** cholesterol (TC) concentration; **(E)** high-density lipoprotein (HDL) concentration; **(F)** low-density lipoprotein (LDL) concentration. Data are presented as means ± SEM. “^*^” represented statistical difference between the NF group and the HF group using student’s *t*-test (^*^ *P* < 0.05, ^**^ *P* < 0.01 or ^***^ *P* < 0.001). “^abc^” represented statistical difference among different RSV levels in the HF diet. *P*-values indicated a linear and quadratic to dietary RSV levels using orthogonal polynomial analysis. Bars with different superscripts are statistically different (*P* < 0.05, n = 3).

The hematological functions parameters of common carp fed with RSV supplementation are shown in **Figure 1**. The ALT and AST activities and the LDL concentration of the HF group were significantly higher than those of the NF group (*P* < 0.05 or *P* < 0.01 or *P* < 0.001), whereas the opposite was found in the HDL concentration (*P* < 0.01). The ALT and AST activities both decreased linearly and quadratically (*P* < 0.001) in response to increasing dietary RSV levels. Additionally, dietary supplementation with RSV decreased linearly (*P* < 0.01) the TG concentration, although there was a weak elevation at the HFR24 group. In addition, the LDL concentrations decreased (*P* < 0.001) with increasing RSV levels, which was accompanied by a quadratic elevation (*P* < 0.001) in the HDL concentration. However, there was no significant difference of TC concentration among all the treatments (*P* > 0.05).

### Hepatic digestive enzyme activities

**Figure 2:**
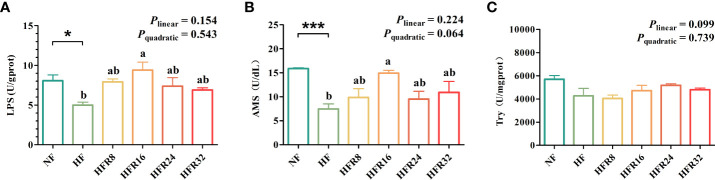
Effects of resveratrol supplementation on the hepatic digestive enzyme activities in common carp (*Cyprinus carpio*). **(A)** lipase (LPS) content; **(B)** amylase (AMS) content; **(C)** trypsin (Try) content. Data are presented as means ± SEM. “^*^” represented statistical difference between the NF group and the HF group using student’s t-test (^*^ *P* < 0.05 or ^***^ *P* < 0.001). “^ab^” represented statistical difference among different RSV levels in the HF diet. *P*-values indicated a linear and quadratic to dietary RSV levels using orthogonal polynomial analysis. Bars with different superscripts are statistically different (*P* < 0.05, n = 3).

The hepatic enzyme activities of common carp fed with RSV supplementation are presented in **Figure 2**. The LPS and AMS activities in the HF diet were significantly lower than those in the NF diet (*P* < 0.05 or *P* < 0.001). However, no linear or quadratic increase (*P* > 0.05) was observed in the LPS and AMS activities, although the HFR16 group had significantly higher LPS and AMS activities than the HF group. Try activity showed no significant difference among all treatments (*P* > 0.05).

### Hepatic antioxidant status

As presented in **Table 4**, the T-SOD and CAT activities and GSH content were significantly lower in fish fed with the HF treatment than those of fish fed by the NF treatment (*P* < 0.05), whereas the MDA content was significantly increased (*P* < 0.05). The T-SOD activity both increased linearly and quadratically (*P* < 0.001) in response to increasing dietary RSV levels, with an opposite result was noted in MDA. A quadratic increase (*P* < 0.001) was observed in the CAT activity, for which declines occurred when dietary RSV levels increased from HFR8 to HFR32. The GSH-Px activity increased quadratically (*P* < 0.001) in response to dietary RSV levels from 0 to 1.6 g/kg, then decreased with further increasing RSV levels. However, GSH content showed no significant difference (*P* > 0.05) among all RSV treatments.

**Table 4:** Effects of resveratrol supplementation on the hepatic antioxidant status in common carp (*Cyprinus carpio*)^a^.

	NF	HF	HFR8	HFR16	HFR24	HFR32	Polynomial contrasts^b^	
							Linear	Quadratic
T-SOD (U·gprot/L)^c^	440.98 ± 24.01	382.65 ± 4.24^d*^	405.43 ± 11.28^c^	475.40 ± 7.43^a^	474.85 ± 5.19^a^	439.80 ± 14.18^b^	0.000	0.000
CAT (μmg·gprot/L)^d^	23.89 ± 3.05	15.52 ± 3.79^d*^	38.73 ± 1.50^a^	30.84 ± 2.51^b^	26.16 ± 1.76^bc^	21.62 ± 2.15^c^	0.937	0.000
GSH (μmol·gprot/L)^e^	74.30 ± 3.51	61.23 ± 4.84^*^	52.63 ± 2.10	59.79 ± 6.29	59.41 ± 2.71	52.04 ± 6.99	0.226	0.643
GSH-Px (μmol·gprot/L)^f^	294.99 ± 8.92	282.70 ± 7.88^b^	288.03 ± 5.68^b^	327.92 ± 14.50^a^	292.78 ± 5.76^b^	286.13 ± 6.38^b^	0.480	0.000
MDA (nmol·gprot/L)^g^	11.96 ± 1.68	14.09 ± 1.79^a*^	10.44 ± 0.59^b^	8.40 ± 0.37^b^	8.71 ± 1.16^b^	9.95 ± 0.49^b^	0.000	0.000

^a^Values expressed as mean ± standard error, n = 3. “^*^” represented statistical difference between the NF group and the HF group using student’s t-test (^*^P < 0.05). “^abcd^” represented statistical difference among different RSV levels in the HF diet. Values in the same row with different superscripts mean significant difference (P < 0.05)

^b^P-values indicated a linear and quadratic to dietary RSV levels using orthogonal polynomial analysis.

^c^T-SOD, total superoxide dismutase;

^d^CAT, catalase;

^e^GSH, glutathione;

^f^GSH-Px, glutathione peroxidase;

^g^MDA, malondialdehyde.

### Hepatic antioxidant-related gene expression

**Figure 3:**
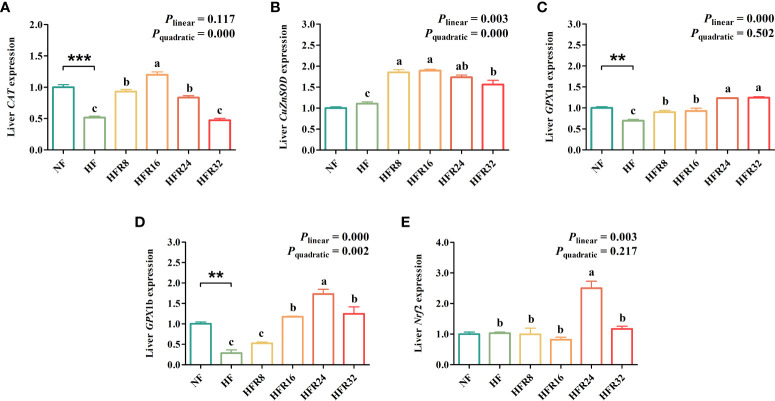
Effects of resveratrol supplementation on the hepatic antioxidant-related gene expression in common carp (*Cyprinus carpio*). **(A)** catalase (CAT); **(B)** copper, zinc superoxide dismutase (CuZnSOD); **(C)** glutathione peroxidase 1a (GPx1a); **(D)** glutathione peroxidase 1b (GPx1b); **(E)** nuclear factor erythroid 2-related factor 2 (Nrf2). Data are presented as means ± SEM. “^*^” represented statistical difference between the NF group and the HF group using student’s t-test (^**^ *P* < 0.01 or ^***^ *P* < 0.001). “^abc^” represented statistical difference among different RSV levels in the HF diet. *P*-values indicated a linear and quadratic to dietary RSV levels using orthogonal polynomial analysis. Bars with different superscripts are statistically different (*P* < 0.05, n = 3).

The hepatic antioxidant-related gene expression of common carp fed with RSV-supplemented diets are exhibited in **Figure 3**. Fish fed with the HF treatment remarkably down-regulated (*P* < 0.01 or *P* < 0.001) the *CAT*, *GPX*1a and *GPX*1b mRNA expression levels relative to fish fed with the NF treatment. The mRNA expression levels of hepatic *CuZnSOD* and *GPX*1b both up-regulated linearly and quadratically (*P *< 0.01 or *P *< 0.001) in response to increasing dietary RSV levels. The mRNA expression level of *CAT* up-regulated quadratically (*P* < 0.001) in response to dietary RSV levels from 0 to 1.6 g/kg, then decreased with further increasing RSV levels. A linear up-regulation (*P *< 0.01 or *P *< 0.001) was noted in the mRNA expression levels of *GPX*1a and *Nrf*2.

### Hepatic lipid metabolism-related gene expression

**Figure 4:**
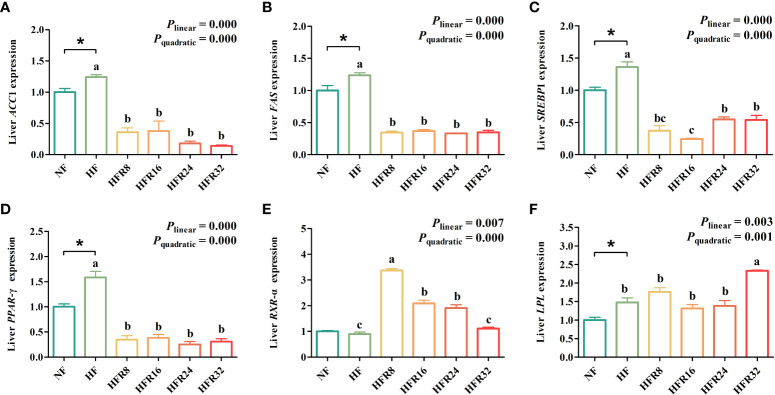
Effects of resveratrol supplementation on the hepatic lipid metabolism-related gene expression in common carp (*Cyprinus carpio*). *de novo* lipogenesis-related genes include **(A)** acetyl-CoA carboxylase 1 (ACC1), **(B)** fatty acid synthase (FAS), and **(C)** peroxisome and sterol regulatory element binding protein 1 (SREBP1). Fatty acid β-oxidation-related genes include **(D)** proliferator activated receptor γ (PPAR-γ) and **(E)** retinoid X receptor α (RXR-α). Fatty acid uptake-related gene includes **(F)** lipase (LPL). Data are presented as means ± SEM. “^*^” represented statistical difference between the NF group and the HF group using student’s t-test (^*^ *P* < 0.05). “^abc^” represented statistical difference among different RSV levels in the HF diet. *P*-values indicated a linear and quadratic to dietary RSV levels using orthogonal polynomial analysis. Bars with different superscripts are statistically different (*P* < 0.05, n = 3).

The hepatic lipid metabolism-related gene expression of common carp fed with RSV-supplemented diets are shown in **Figure 4**. Fish fed with the HF treatment significantly up-regulated (*P* < 0.05) the mRNA expression levels of *ACC*1, *FAS*, *SREBP*1, *PPAR*-γ, and *LPL* relative to fish fed with the NF treatment. Simultaneously, the mRNA expression levels *ACC*1, *FAS*, *SREBP*1, and *PPAR*-γ both down-regulated linearly and quadratically (*P *< 0.001) in response to increasing dietary RSV levels. A pronounced up-regulation was observed for mRNA expression level of *RXR*-α with increasing RSV levels (linear effect, P =0.007 or quadratic effect, P < 0.001). Additionally, the expression mRNA level of LPL up-regulated linearly and quadratically (*P *< 0.01 or *P *< 0.001) in response to increasing dietary RSV levels.

### Hepatic inflammatory cytokines-related gene expression

**Figure 5:**
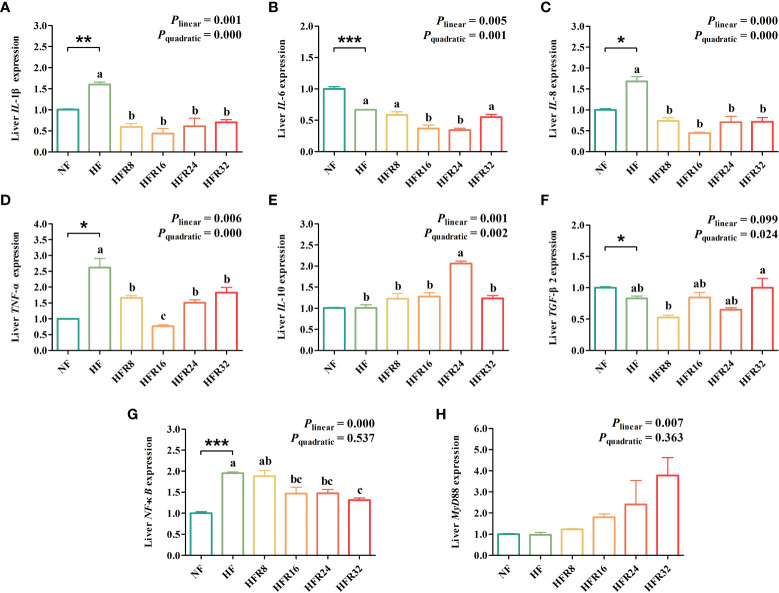
Effects of resveratrol supplementation on the hepatic lipid metabolism-related gene expression in common carp (*Cyprinus carpio*). Pro-inflammatory-related genes include **(A)** interleukin-1β (IL-1β), **(B)** interleukin-6 (IL-6), **(C)** interleukin-8 (IL-8), and **(D)** tumor necrosis factor-α (TNF-α). Anti-inflammatory-related genes **(E)** interleukin-10 (IL-10) and **(F)** transforming growth factor-β2 (TGF-β2). Signaling pathways include **(G)** nuclear factor kappa-B (NF-κB) and **(H)** myeloid differentiation factor 88 (MyD88). Data are presented as means ± SEM. “^*^” represented statistical difference between the NF group and the HF group using student’s t-test (^*^ *P* < 0.05, ^**^ *P* < 0.01 or ^***^ *P* < 0.001). “^abc^” represented statistical difference among different RSV levels in the HF diet. *P*-values indicated a linear and quadratic to dietary RSV levels using orthogonal polynomial analysis. Bars with different superscripts are statistically different (*P* < 0.05, n = 3).

As presented in **Figure 5**, the results showed that the mRNA expression levels of *IL*-1β, *IL*-8, *TNF*-α, and *NF*-κB were significantly up-regulated (*P* < 0.05 or *P* < 0.01 or *P* < 0.001) in the liver of common carp fed with the HF diet compared with the NF diet, whereas an opposite result was noted in *IL*-6 and *TGF*-β2. Additionally, the mRNA expression levels *IL*-1β, *IL*-6, *IL*-8, and *TNF*-α both down-regulated linearly and quadratically (*P* < 0.01 or *P *< 0.001) in response to increasing dietary RSV levels. A pronounced up-regulation was observed for mRNA expression level of *IL*-10 with increasing RSV levels (linear effect, P =0.001 or quadratic effect, P =0.002). The mRNA expression level of *TGF*-β2 down-regulated quadratically (*P* < 0.01) in response to dietary RSV levels from 0 to 0.8 g/kg, then decreased with further increasing RSV levels. A linear down-regulation (*P* < 0.001) was observed in the mRNA expression level of *NF-κB*. However, an opposite result was noted in *MyD*88 (*P* < 0.01).

### Dietary RSV requirements for common carp in an HF diet

**Figure 6:**
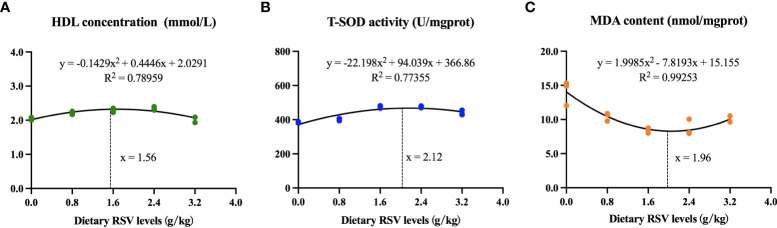
Dietary RSV requirements for common carp based on **(A)** high-density lipoprotein (HDL) concentration, **(B)** total superoxide dismutase (T-SOD) activity and **(C)** malondialdehyde (MDA) content.

As shown in **Figure 6**, based on HDL concentration, T-SOD activity, and MDA content, the dietary RSV requirements for common carp were determined to be 1.56 g/kg and 2.12 g/kg in an HF diet, respectively.

## Discussion

Lipid is an important nutrient, which provides the energy required for growth and development of aquatic animals. Moderate lipid levels have the dual effect of sparing protein and facilitating the absorption of other nutrients. However, fish has caused problems such as slowed growth rate, excessive deposition of body fat, and decreased anti-stress ability after consuming an HF diet. Previous studies showed that HF diet impaired the growth performance of Pacific white shrimp (*Litopenaeus vannamei*) (20), blunt snout bream (21), spotted seabass (*Lateolabrax maculatus*) (22), and largemouth bass (*Micropterus salmoides*) (23). The present study indicated that, compared with the NF diet, the HF diet provoked an overt weight extremes characterized by the decreased FBW and WGR. Even more importantly, the HSI was increased by HF diet feeding, indicating that lipid transport and amount of fat deposition was affected by the HF diet. Our data also confirmed that the HF diet induced liver damage evidenced by the elevated hematological functions parameters. Additionally, oxidative stress, increased pro-inflammatory cytokine production, and increased expression levels of genes related to lipid metabolism in liver of carp fed with HF diet were also observed. These data indicated that the liver damage model was successfully established. As expected, aforementioned results including the FBW, WGR and HSI were reversed by RSV treatment in different levels, suggesting that RSV ameliorated the liver damage in carp induced by HF diet partly due to the improvement of redox, lipid metabolism level and immune status. Similar results have been observed in flounder (*Paralichthys lethostigma*) (24) and tilapia (*Oreochromis niloticus*) (25). The growth enhancement was attributed to the depression of protein oxidative damage and ubiquitinylation (24). Some studies demonstrated that an inhibitory growth performance was found in blunt snout bream fed with 1.08% RSV, which may be related to animal appetite (26). RSV has been shown to inhibit neuropeptide Y (NPY) expression and lead to a reduction in intake (27). The above results are contrary to our data, which may be due to the fact that the supplementation of RSV added in our study was not enough to inhibit growth. It is worth noting that, in our study, the supplementation of 3.2 g/kg RSV in the HF diet increased the FBW of carp, but also led to an increase in the HSI of carp. Therefore, the supplemental content of 3.2 g/kg RSV was not the most appropriate choice.

The improvement of digestive enzyme activity is beneficial to the digestion and absorption of nutrients by fish, thus accelerating the growth and development (28). A previous study in largemouth bass showed that dietary HF diet may reduce the digestive enzyme activities of the organisms, which was consistent with the results of this study that feeding HF diet significantly reduced the activities of LPS, AMS, and Try in the liver of carp (23). In Nile tilapia, dietary supplementation of 50 mg/kg RSV improved hepatopancreas digestive capacity (29). Similarly, our current results indicated that the digestion of carp supplemented with 1.6 g/kg RSV was consistent with that of NF group, indicating that RSV could maintain growth by increasing digestive enzyme activity. However, the specific mechanism of RSV regulating digestive enzymes remains to be elucidated.

In general, liver damage is usually accompanied by increased serum AST and ALT activities, and the TC and TG concentrations are the reflection of lipid metabolism. Here, the increased ALT and AST activities in the serum of carp fed with an HF diet indicated the impaired function and metabolism was appeared in the liver, and increased serum TC concentration in the HF group also suggested lipid metabolism disorders, which may lead to oxidative stress and reduced disease resistance. Previous studies has showed that the HF diet had a remarkable effect on blood biochemistry in blunt snout bream (30) and grass carp (31). Data in our study suggested that dietary RSV supplementation decreased the ALT and AST activities, as well as TG concentration in carp. As a result, our study were agreement with previous study, which demonstrated that the appropriate amounts of dietary RSV improves the metabolic health in Nile tilapia (13) and rats (32). RSV had also beneficial effects on lipid profile, considering the importance of HDL to the reverse transport of cholesterol. Note that RSV supplementation increased the HDL content, as well as decreased the LDL content, which were significant risk factors related to fatty liver. Overall, this is consistent with the known data on the lipid-lowering effects of RSV from several previous studies (33, 34). On the other hand, digestive enzyme activity is considered as an index to evaluate the digestion and absorption ability of fish, reflecting the response of fish to environmental changes. In the present study, HF diet reduced the LPS and AMS activities in liver, which to some extent restricted the digestive function of carp. Moreover, with the supplementation of dietary RSV, the LPS and AMS activities increased and then decreased and the highest values were appeared in the HFR16 group, which may be due to the fact that RSV activated lipogenic enzymes in the liver and bound to the surface of TG, catalyzing the digestion and utilization of lipids. Thus, these results indicated that appropriate dose of RSV could inhibit HF diet-induced hepatic damage and had a benefit for maintaining health.

High ROS production caused by HF diet causes oxidative stress in organisms (35). In the present study, the increased MDA content and reduced T-SOD activity were decreased in the HF diet. Similar results were observed in largemouth bass (36) and blunt snout bream (37). MDA is a biomarker of lipid peroxidation in animal tissues, and T-SOD is the main direct response of antioxidant defense against ROS (35). This indicates that high level of fat might induce oxidative damage and lipid peroxidation. In addition, organisms rely on an antioxidant protection system to prevent oxidative injury to prevent metabolic stress caused by an HF diet (38). The inhibition of the antioxidant enzyme system composed of CAT activity and GSH content in the HF diet further supported the notion that the HF diet induced oxidative stress in our study. In fact, there is increasing evidence that HF dietary intake leads to higher ROS production, which induces oxidative stress and lipid hydrogen peroxide formation (39). Judging from our data, a notable finding of our study was that RSV enhanced the T-SOD, CAT, and GSH-Px activities, as well as reduced the MDA content in the carp, indicating that RSV supplementation had distinct effects on hepatic markers of oxidative stress. These results were similar to pervious study in which GSH content increased and then decreased with increasing amount of RSV supplementation, Moreover, CAT activity in liver was the highest at 0.8 g/kg RSV supplementation in this study. This result was different from the previous studies in which the serum CAT activity in black-boned chickens (40) and the liver CAT viability in tilapia (25) reached a maximum at 0.4 g/kg and 0.6 g/kg RSV supplementation, respectively. This may be related to species and tissue distribution and RSV may act differently in different concentration ranges. RSV had a biphasic effect on the activation and inhibition of antioxidant enzymes (40). Anyway, according to our experimental results, we could affirm that ROS was scavenged by appropriate dose of RSV, thus reducing the hepatic oxidative damage and improving the antioxidant defense in fish. As previously reported, RSV has a strong ROS scavenging activity due to the presence of two phenol rings and multiple hydroxyl groups which act as H atom donor (41). A dietary RSV would offer H atom donors to enhance the scavenging capacity of ROS.

The expression of antioxidant enzyme genes is regulated by multiple transcription factors. Nrf2 is a key transcription factor in the endogenous antioxidant defense system, which essential for protecting against liver injury induced by oxidative stress (42). In our current study, the gene expression of *Nrf*2 showed no significant up-regulation in the HF diet. Previous study has confirmed that gene expression of Nrf2 is suppressed under severe oxidative stress (43, 44). Our results possibly imputed to dysfunction of Nrf2 system induced by severe oxidative stress, and reflected cell damage and depression of antioxidant status (45). Additionally, it was also proved that the gene expression levels of CAT, GPx1a and GPx1b were all decreased compared with the NF group in our study, suggesting that the oxidative stress occurred in carp caused by HF diet. A previous study has proved that RSV was well described to trigger the Nrf2 pathway which promotes the transcription of antioxidant-related genes (46). In the present study, dietary RSV resulted in an increase in *CAT*, *CuZnSOD*, *GPx*1a and *GPx*1b expression levels in liver, which might partly explain the increased activities of *T-SOD*, *CAT* and *GSH-Px*. Similar to our results for antioxidant-active enzymes, the current study found that the expression levels of *CAT*, *CuZnSOD*, *GPX1b* and *Nrf2* were increased and then decreased after supplementation with different levels of RSV. This implied that RSV regulated antioxidant enzyme activity through antioxidant gene expression levels and exhibited stimulatory effects at appropriate doses. These findings were consistent with a previous report in which RSV inhibited the degradation of Nrf2 protein mediated by ubiquitin, thereby stabilizing the concentration of Nrf2 protein in the cytoplasm, enhancing the expression of genes such as Nrf2 downstream antioxidant proteins (SOD, GSH-Px) and phase II detoxification enzymes under stress conditions, thereby improving antioxidant capacity of organisms (46).

The continuous development of oxidative stress is bound to aggravate the inflammatory response. Oxidative stress activates a variety of transcription factors, resulting in differential expression of genes associated with inflammatory pathways (47). Pro-inflammatory factors, such as IL-1β, IL-6, and TNF-α are early markers of chronic inflammation in organisms, leading to liver damage (48). IL-1β induces the expression of various pro-inflammatory genes, resulting in the accumulation of triacylglycerols, steatohepatitis and fibrosis. In addition, IL-1β also induces hepatocyte death by inhibiting the expression of fatty acid synthases and enhancing the expression of TNF-α (49). IL-6 is involved in immune regulation and lipid metabolism, and its high expression is usually associated with obesity and insulin resistance. TNF-α is involved in the regulation of immune response, inflammatory response and lipid metabolism, and is an important cytokine that causes liver damage (50). The results of our study showed that the expression levels of *IL*⁃1β and *TNF*⁃α in liver of the HF group were higher than those of the NF group, indicating that the HF diet stimulated the imbalance of the proportion of inflammatory cytokines in carp, leading to inflammation. Growing evidence has suggested that the HF diets not only cause dyslipidemia, but also keep the organism in a chronic state of inflammation for a long time (51). Previous research confirmed that reducing inflammation might ameliorate the insulin sensitivity and decrease the hepatic parameters reflecting liver damage (52). It was somewhat surprising that the expression level of *IL-6* was down-regulated in the HF group. This finding was consistent with previous study which have suggested that HF diet reduced serum IL-6 concentration in mice (53) Judging from our findings, RSV supplementation down-regulated the gene expression of pro-inflammatory factors in liver. These results demonstrated that RSV supplementation attenuated hepatic inflammation in the HF diet. Similar results were obtained in a previous study showing that RSV improved the pro-inflammatory profile of mice associated with lipogenesis under an HF challenge by suppressing the expression of TNF-α, IL-6, and NF-κB (54). As an anti-inflammatory cytokine, IL-10 antagonizes the pro-inflammatory effects of other cytokines, thereby controlling inflammation. IL-10 has been shown to inhibit the expression of cytokines such as TNF-α and IL-1β (55), which is consistent with the results of this study. In terms of mechanism, RSV could up-regulate the expression of Sirt1 in tissues or cells to a certain extent, and then regulate inflammatory factors and related signal transduction pathways to inhibit inflammatory responses (56). One unanticipated finding was that MyD88 gene expression level was gradually upregulated with increasing RSV supplementation, while the differences between groups were not significant. Another finding of the current study was that TNF-α gene expression level was upregulated after dietary supplementation with high doses of RSV. Consistent with our finding, the result of the previous study indicated that liver tissue significantly damaged in the high-dose RSV group (57). Previous findings showed that liver tissue was significantly damaged in the high-dose RES group. TNF-α further increased intestinal permeability and induced apoptosis and production of other cytokines, which led to persistent worsening of liver injury. Hepatocyte necrosis and apoptosis in the high-dose RES group may be due to intestinal absorption of RES leading to liver injury. The lower dose of RSV, on the other hand, was associated with enhanced immunity (58).

The hepatic lipid metabolism is comprehensively regulated by a variety of genes related to liver oxidation, transport and uptake of lipids (59). The hepatic release of fatty acid uptake from circulating triacylglycerol, fatty acid oxidation, and *de novo* lipogenesis, and lipolysis are key metabolic pathways that control hepatic triacylglycerol metabolism, which play a crucial role in the development of liver steatosis (60). In our study, the mRNA levels of the above-mentioned metabolic pathway-related genes were used to evaluate the lipid metabolism mechanism of RSV. With regard to enzymes related to hepatic *de novo* lipogenesis, a significant up-regulation in *SREBP*1, *ACC*1, and *FAS* was observed in the HF diet. As a rate-limiting enzyme in the *de novo* adipogenesis pathway, ACC catalyzes the carboxylation of acetyl-CoA to malonyl-CoA (61). Similarly, FAS is responsible for catalyzing the whole process of malonyl-CoA synthesis of palmitate and plays a key role in the *de novo* fatty acid synthesis pathway (62). Our data indicated that the HF diet in carp promoted the fat deposition in liver, and above results are also in good accordance with the increase of MDA content in the HF diet. A remarkably lower expression of *ACC*1 and *FAS* was observed in the carp fed with the RSV-supplemented diets, showing that RSV decreased lipogenesis. Previous studies in mice showed that the expression levels of ACC and FAS were decreased by RSV (63). Expectedly, we demonstrated that RSV led to a decreased expression level of *SREBP*1. Down-regulation of SREBP1 decreases the uptake and absorption of glycerol by hepatocytes and inhibits the synthesis of fatty acids and cholesterol (64). This is also partly consistent with the decrease of TG concentration. As for lipolysis, LPL is the key enzyme responsible for hydrolysis of triacylglycerol (65). RSV supplementation with 1.6 g/kg and 2.4 g/kg decreased the expression levels of *LPL* in our study. These results are consistent with a previous study in rats (63), which suggested that the fat-lowing effect of RSV might be partly mediated by an enhancement in lipolysis capacity. With regard to fatty acid oxidation, the expression of *PPAR*-γ, a transcription factor which can upregulate the genes expression of key proteins involved in fatty acid β-oxidation, was increased in the HF diet, whereas RSV decreased the expression levels of PPAR-γ in liver.

In view of the foregoing, the lipid accumulation in the liver by HF diet is achieved through the increased lipogenesis and fatty acid uptake from circulation, which may contribute to the increased oxidative stress in the liver. RSV alleviates liver damage and oxidative stress induced by HF diet through its antioxidant properties and the activation of the antioxidant enzyme system *via* Nrf2/NF-κB/PPAR-γ signaling pathway. The supplemented RSV requirements for ameliorating oxidative stress, inflammatory response and lipid metabolism of common carp were determined to be 1.56 - 2.12 g/kg.

## Data availability statement

The original contributions presented in the study are included in the article/supplementary material. Further inquiries can be directed to the corresponding authors.

## Ethics statement

The animal study was reviewed and approved by the Committee for the Welfare and Ethics of Laboratory Animals of Heilongjiang River Fisheries Research Institute of Chinese Academy of Fishery Sciences.

## Author contributions

Conceptualization: DW and LW. Methodology: DW and JL. Data curation: DW. Writing—original draft preparation: DW. Writing—review and editing: JL, ZF, and LW. Funding acquisition: DW, LW and XZ. All authors contributed to the article and approved the submitted version.

## Funding

This work was supported by the Central Public-interest Scientific Institution Basal Research Fund, HRFRI (HSY202206Q), the Central Public-interest Scientific Institution Basal Research Fund, CAFS (2022XT0402), the Natural Science Foundation of China, NSFC (32072972), and the China Agriculture Research System of MOF and MARA (CARS-45).

## Conflict of interest

The authors declare that the research was conducted in the absence of any commercial or financial relationships that could be construed as a potential conflict of interest.

## Publisher’s note

All claims expressed in this article are solely those of the authors and do not necessarily represent those of their affiliated organizations, or those of the publisher, the editors and the reviewers. Any product that may be evaluated in this article, or claim that may be made by its manufacturer, is not guaranteed or endorsed by the publisher.
